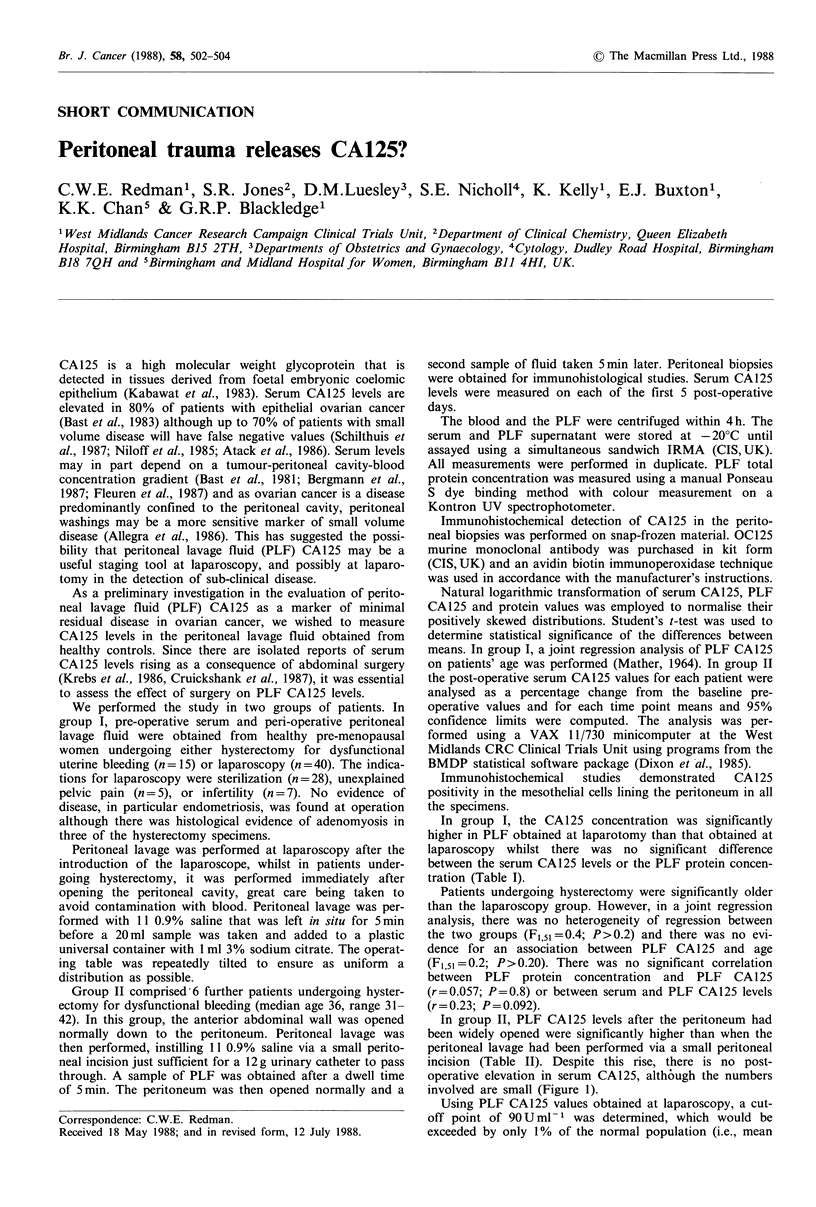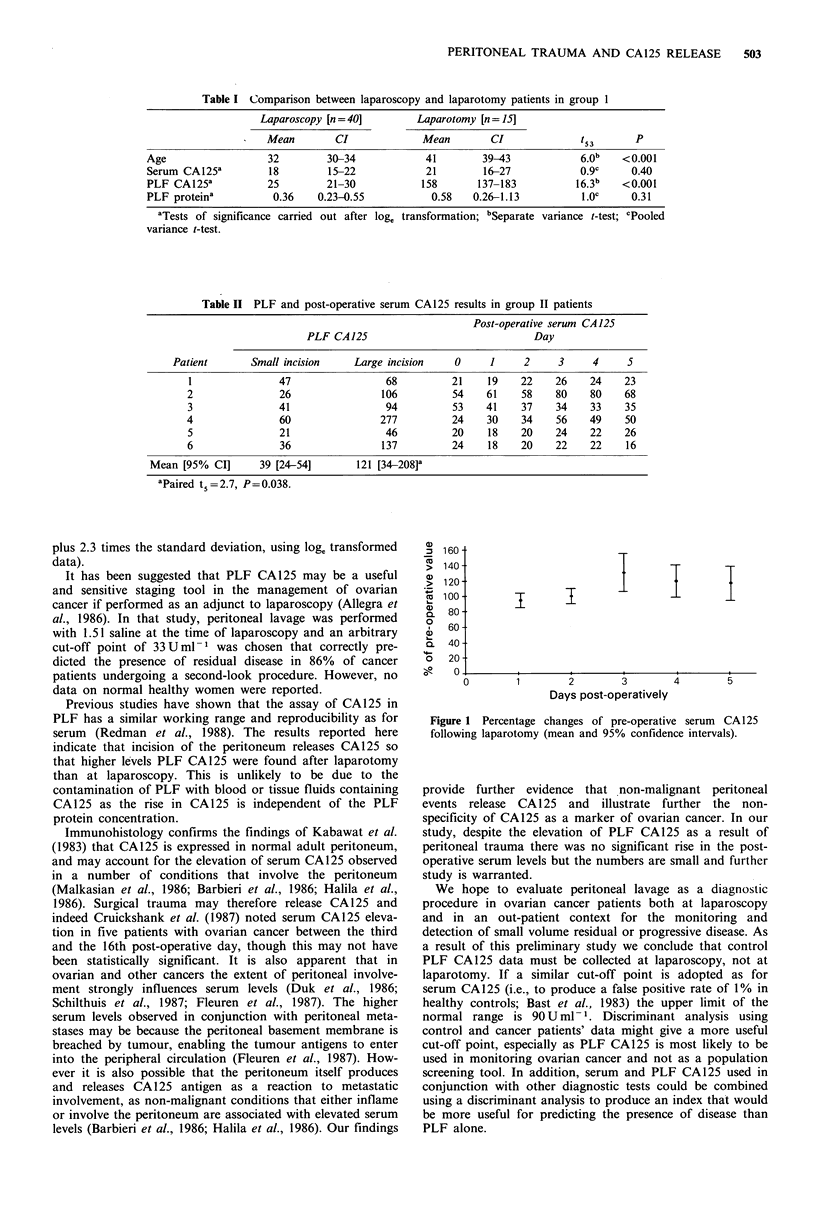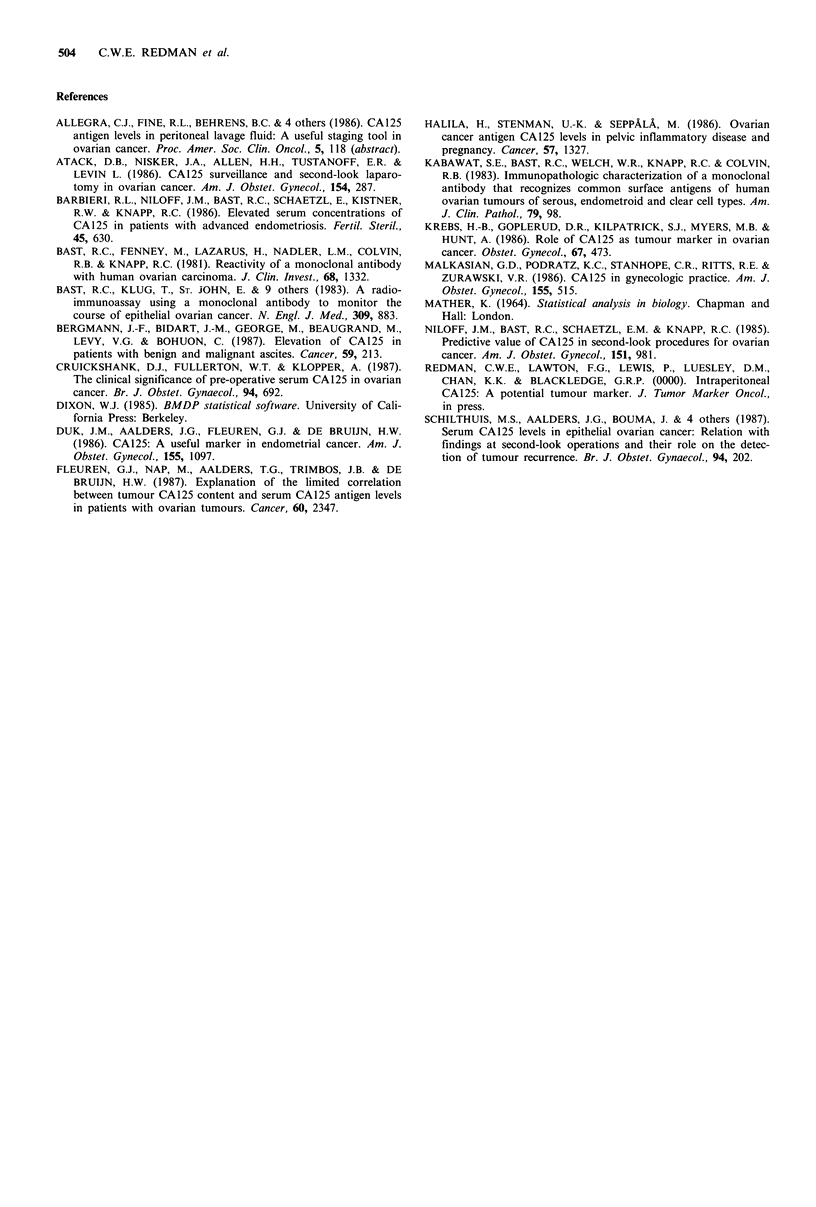# Peritoneal trauma releases CA125?

**DOI:** 10.1038/bjc.1988.250

**Published:** 1988-10

**Authors:** C. W. Redman, S. R. Jones, D. M. Luesley, S. E. Nicholl, K. Kelly, E. J. Buxton, K. K. Chan, G. R. Blackledge

**Affiliations:** West Midlands Cancer Research Campaign Clinical Trials Unit, Queen Elizabeth Hospital, Birmingham, UK.


Br. J. Cancer (1988), 58, 502–504 © The Macmillan Press Ltd., 1988

SHORT COMMUNICATION

### Peritoneal trauma releases CA125?

C.W.E. Redman[1], S.R. Jones[2], D.M.Luesley[3], S.E. Nicholl[4], K. Kelly[1], E.J. Buxton[1], K.K. Chan[5] & G.R.P. Blackledge[1]

[1]*West Midlands Cancer Research Campaign Clinical Trials Unit,* [2]*Department of Clinical Chemistry, Queen Elizabeth Hospital, Birmingham B15 2TH,* [3]*Departments of Obstetrics and Gynaecology,* [4]*Cytology, Dudley Road Hospital, Birmingham B18 7QH and* [5]*Birmingham and Midland Hospital for Women, Birmingham B11 4HI, UK.*

CA125 is a high molecular weight glycoprotein that is detected in tissues derived from foetal embryonic coelomic epithelium (Kabawat *et al.*, 1983). Serum CA125 levels are elevated in 80% of patients with epithelial ovarian cancer (Bast *et al.*, 1983) although up to 70% of patients with small volume disease will have false negative values (Schilthuis *et al.*, 1987; Niloff *et al.*, 1985; Atack *et al.*, 1986). Serum levels may in part depend on a tumour-peritoneal cavity-blood concentration gradient (Bast *et al.*, 1981; Bergmann *et al.*, 1987; Fleuren *et al.*, 1987) and as ovarian cancer is a disease predominantly confined to the peritoneal cavity, peritoneal washings may be a more sensitive marker of small volume disease (Allegra *et al.*, 1986). This has suggested the possibility that peritoneal lavage fluid (PLF) CA125 may be a useful staging tool at laparoscopy, and possibly at laparotomy in the detection of sub-clinical disease.

As a preliminary investigation in the evaluation of peritoneal lavage fluid (PLF) CA125 as a marker of minimal residual disease in ovarian cancer, we wished to measure CA125 levels in the peritoneal lavage fluid obtained from healthy controls. Since there are isolated reports of serum CA125 levels rising as a consequence of abdominal surgery (Krebs *et al.*, 1986, Cruickshank *et al.*, 1987), it was essential to assess the effect of surgery on PLF CA125 levels.

We performed the study in two groups of patients. In group I, pre-operative serum and peri-operative peritoneal lavage fluid were obtained from healthy pre-menopausal women undergoing either hysterectomy for dysfunctional uterine bleeding ($n=15$) or laparoscopy ($n=40$). The indications for laparoscopy were sterilization ($n=28$), unexplained pelvic pain ($n=5$), or infertility ($n=7$). No evidence of disease, in particular endometriosis, was found at operation although there was histological evidence of adenomyosis in three of the hysterectomy specimens.

Peritoneal lavage was performed at laparoscopy after the introduction of the laparoscope, whilst in patients undergoing hysterectomy, it was performed immediately after opening the peritoneal cavity, great care being taken to avoid contamination with blood. Peritoneal lavage was performed with 1 l 0.9% saline that was left *in situ* for 5 min before a 20 ml sample was taken and added to a plastic universal container with 1 ml 3% sodium citrate. The operating table was repeatedly tilted to ensure as uniform a distribution as possible.

Group II comprised 6 further patients undergoing hysterectomy for dysfunctional bleeding (median age 36, range 31–42). In this group, the anterior abdominal wall was opened normally down to the peritoneum. Peritoneal lavage was then performed, instilling 1 l 0.9% saline via a small peritoneal incision just sufficient for a 12 g urinary catheter to pass through. A sample of PLF was obtained after a dwell time of 5 min. The peritoneum was then opened normally and a second sample of fluid taken 5 min later. Peritoneal biopsies were obtained for immunohistological studies. Serum CA125 levels were measured on each of the first 5 post-operative days.

The blood and the PLF were centrifuged within 4 h. The serum and PLF supernatant were stored at −20°C until assayed using a simultaneous sandwich IRMA (CIS, UK). All measurements were performed in duplicate. PLF total protein concentration was measured using a manual Ponseau S dye binding method with colour measurement on a Kontron UV spectrophotometer.

Immunohistochemical detection of CA125 in the peritoneal biopsies was performed on snap-frozen material. OC125 murine monoclonal antibody was purchased in kit form (CIS, UK) and an avidin biotin immunoperoxidase technique was used in accordance with the manufacturer's instructions.

Natural logarithmic transformation of serum CA125, PLF CA125 and protein values was employed to normalise their positively skewed distributions. Student's *t*-test was used to determine statistical significance of the differences between means. In group I, a joint regression analysis of PLF CA125 on patients' age was performed (Mather, 1964). In group II the post-operative serum CA125 values for each patient were analysed as a percentage change from the baseline pre-operative values and for each time point means and 95% confidence limits were computed. The analysis was performed using a VAX 11/730 minicomputer at the West Midlands CRC Clinical Trials Unit using programs from the BMDP statistical software package (Dixon *et al.*, 1985).

Immunohistochemical studies demonstrated CA125 positivity in the mesothelial cells lining the peritoneum in all the specimens.

In group I, the CA125 concentration was significantly higher in PLF obtained at laparotomy than that obtained at laparoscopy whilst there was no significant difference between the serum CA125 levels or the PLF protein concentration (Table I).

Patients undergoing hysterectomy were significantly older than the laparoscopy group. However, in a joint regression analysis, there was no heterogeneity of regression between the two groups ($F_{1,51}=0.4$; $P>0.2$) and there was no evidence for an association between PLF CA125 and age ($F_{1,51}=0.2$; $P>0.20$). There was no significant correlation between PLF protein concentration and PLF CA125 ($r=0.057$; $P=0.8$) or between serum and PLF CA125 levels ($r=0.23$; $P=0.092$).

In group II, PLF CA125 levels after the peritoneum had been widely opened were significantly higher than when the peritoneal lavage had been performed via a small peritoneal incision (Table II). Despite this rise, there is no post-operative elevation in serum CA125, although the numbers involved are small (Figure 1).

Using PLF CA125 values obtained at laparoscopy, a cut-off point of 90 U ml$^{-1}$ was determined, which would be exceeded by only 1% of the normal population (i.e., mean

Correspondence: C.W.E. Redman.

Received 18 May 1988; and in revised form, 12 July 1988.

**Table I** Comparison between laparoscopy and laparotomy patients in group 1

| | *Laparoscopy* [$n=40$] | | *Laparotomy* [$n=15$] | | | |
|---|---|---|---|---|---|---|
| | *Mean* | *CI* | *Mean* | *CI* | $t_{53}$ | *P* |
| Age | 32 | 30–34 | 41 | 39–43 | 6.0[b] | <0.001 |
| Serum CA125[a] | 18 | 15–22 | 21 | 16–27 | 0.9[c] | 0.40 |
| PLF CA125[a] | 25 | 21–30 | 158 | 137–183 | 16.3[b] | <0.001 |
| PLF protein[a] | 0.36 | 0.23–0.55 | 0.58 | 0.26–1.13 | 1.0[c] | 0.31 |

[a]Tests of significance carried out after $\log_e$ transformation; [b]Separate variance *t*-test; [c]Pooled variance *t*-test.

**Table II** PLF and post-operative serum CA125 results in group II patients

| | *PLF CA125* | | *Post-operative serum CA125 Day* | | | | | |
|---|---|---|---|---|---|---|---|---|
| *Patient* | *Small incision* | *Large incision* | *0* | *1* | *2* | *3* | *4* | *5* |
| 1 | 47 | 68 | 21 | 19 | 22 | 26 | 24 | 23 |
| 2 | 26 | 106 | 54 | 61 | 58 | 80 | 80 | 68 |
| 3 | 41 | 94 | 53 | 41 | 37 | 34 | 33 | 35 |
| 4 | 60 | 277 | 24 | 30 | 34 | 56 | 49 | 50 |
| 5 | 21 | 46 | 20 | 18 | 20 | 24 | 22 | 26 |
| 6 | 36 | 137 | 24 | 18 | 20 | 22 | 22 | 16 |
| Mean [95% CI] | 39 [24–54] | 121 [34–208][a] | | | | | | |

[a]Paired $t_5=2.7$, $P=0.038$.

plus 2.3 times the standard deviation, using $\log_e$ transformed data).

It has been suggested that PLF CA125 may be a useful and sensitive staging tool in the management of ovarian cancer if performed as an adjunct to laparoscopy (Allegra *et al.*, 1986). In that study, peritoneal lavage was performed with 1.5 l saline at the time of laparoscopy and an arbitrary cut-off point of 33 U ml$^{-1}$ was chosen that correctly predicted the presence of residual disease in 86% of cancer patients undergoing a second-look procedure. However, no data on normal healthy women were reported.

Previous studies have shown that the assay of CA125 in PLF has a similar working range and reproducibility as for serum (Redman *et al.*, 1988). The results reported here indicate that incision of the peritoneum releases CA125 so that higher levels PLF CA125 were found after laparotomy than at laparoscopy. This is unlikely to be due to the contamination of PLF with blood or tissue fluids containing CA125 as the rise in CA125 is independent of the PLF protein concentration.

Immunohistology confirms the findings of Kabawat *et al.* (1983) that CA125 is expressed in normal adult peritoneum, and may account for the elevation of serum CA125 observed in a number of conditions that involve the peritoneum (Malkasian *et al.*, 1986; Barbieri *et al.*, 1986; Halila *et al.*, 1986). Surgical trauma may therefore release CA125 and indeed Cruickshank *et al.* (1987) noted serum CA125 elevation in five patients with ovarian cancer between the third and the 16th post-operative day, though this may not have been statistically significant. It is also apparent that in ovarian and other cancers the extent of peritoneal involvement strongly influences serum levels (Duk *et al.*, 1986; Schilthuis *et al.*, 1987; Fleuren *et al.*, 1987). The higher serum levels observed in conjunction with peritoneal metastases may be because the peritoneal basement membrane is breached by tumour, enabling the tumour antigens to enter into the peripheral circulation (Fleuren *et al.*, 1987). However it is also possible that the peritoneum itself produces and releases CA125 antigen as a reaction to metastatic involvement, as non-malignant conditions that either inflame or involve the peritoneum are associated with elevated serum levels (Barbieri *et al.*, 1986; Halila *et al.*, 1986). Our findings provide further evidence that non-malignant peritoneal events release CA125 and illustrate further the non-specificity of CA125 as a marker of ovarian cancer. In our study, despite the elevation of PLF CA125 as a result of peritoneal trauma there was no significant rise in the post-operative serum levels but the numbers are small and further study is warranted.

**Figure 1** Percentage changes of pre-operative serum CA125 following laparotomy (mean and 95% confidence intervals).

We hope to evaluate peritoneal lavage as a diagnostic procedure in ovarian cancer patients both at laparoscopy and in an out-patient context for the monitoring and detection of small volume residual or progressive disease. As a result of this preliminary study we conclude that control PLF CA125 data must be collected at laparoscopy, not at laparotomy. If a similar cut-off point is adopted as for serum CA125 (i.e., to produce a false positive rate of 1% in healthy controls; Bast *et al.*, 1983) the upper limit of the normal range is 90 U ml$^{-1}$. Discriminant analysis using control and cancer patients' data might give a more useful cut-off point, especially as PLF CA125 is most likely to be used in monitoring ovarian cancer and not as a population screening tool. In addition, serum and PLF CA125 used in conjunction with other diagnostic tests could be combined using a discriminant analysis to produce an index that would be more useful for predicting the presence of disease than PLF alone.

### References

ALLEGRA, C.J., FINE, R.L., BEHRENS, B.C. & 4 others (1986). CA125 antigen levels in peritoneal lavage fluid: A useful staging tool in ovarian cancer. *Proc. Amer. Soc. Clin. Oncol.*, **5**, 118 (*abstract*).

ATACK, D.B., NISKER, J.A., ALLEN, H.H., TUSTANOFF, E.R. & LEVIN L. (1986). CA125 surveillance and second-look laparotomy in ovarian cancer. *Am. J. Obstet. Gynecol.*, **154**, 287.

BARBIERI, R.L., NILOFF, J.M., BAST, R.C., SCHAETZL, E., KISTNER, R.W. & KNAPP, R.C. (1986). Elevated serum concentrations of CA125 in patients with advanced endometriosis. *Fertil. Steril.*, **45**, 630.

BAST, R.C., FENNEY, M., LAZARUS, H., NADLER, L.M., COLVIN, R.B. & KNAPP, R.C. (1981). Reactivity of a monoclonal antibody with human ovarian carcinoma. *J. Clin. Invest.*, **68**, 1332.

BAST, R.C., KLUG, T., ST. JOHN, E. & 9 others (1983). A radioimmunoassay using a monoclonal antibody to monitor the course of epithelial ovarian cancer. *N. Engl. J. Med.*, **309**, 883.

BERGMANN, J.-F., BIDART, J.-M., GEORGE, M., BEAUGRAND, M., LEVY, V.G. & BOHUON, C. (1987). Elevation of CA125 in patients with benign and malignant ascites. *Cancer*, **59**, 213.

CRUICKSHANK, D.J., FULLERTON, W.T. & KLOPPER, A. (1987). The clinical significance of pre-operative serum CA125 in ovarian cancer. *Br. J. Obstet. Gynaecol.*, **94**, 692.

DIXON, W.J. (1985). *BMDP statistical software*. University of California Press: Berkeley.

DUK, J.M., AALDERS, J.G., FLEUREN, G.J. & DE BRUIJN, H.W. (1986). CA125: A useful marker in endometrial cancer. *Am. J. Obstet. Gynecol.*, **155**, 1097.

FLEUREN, G.J., NAP, M., AALDERS, T.G., TRIMBOS, J.B. & DE BRUIJN, H.W. (1987). Explanation of the limited correlation between tumour CA125 content and serum CA125 antigen levels in patients with ovarian tumours. *Cancer*, **60**, 2347.

HALILA, H., STENMAN, U.-K. & SEPPÅLÅ, M. (1986). Ovarian cancer antigen CA125 levels in pelvic inflammatory disease and pregnancy. *Cancer*, **57**, 1327.

KABAWAT, S.E., BAST, R.C., WELCH, W.R., KNAPP, R.C. & COLVIN, R.B. (1983). Immunopathologic characterization of a monoclonal antibody that recognizes common surface antigens of human ovarian tumours of serous, endometroid and clear cell types. *Am. J. Clin. Pathol.*, **79**, 98.

KREBS, H.-B., GOPLERUD, D.R., KILPATRICK, S.J., MYERS, M.B. & HUNT, A. (1986). Role of CA125 as tumour marker in ovarian cancer. *Obstet. Gynecol.*, **67**, 473.

MALKASIAN, G.D., PODRATZ, K.C., STANHOPE, C.R., RITTS, R.E. & ZURAWSKI, V.R. (1986). CA125 in gynecologic practice. *Am. J. Obstet. Gynecol.*, **155**, 515.

MATHER, K. (1964). *Statistical analysis in biology*. Chapman and Hall: London.

NILOFF, J.M., BAST, R.C., SCHAETZL, E.M. & KNAPP, R.C. (1985). Predictive value of CA125 in second-look procedures for ovarian cancer. *Am. J. Obstet. Gynecol.*, **151**, 981.

REDMAN, C.W.E., LAWTON, F.G., LEWIS, P., LUESLEY, D.M., CHAN, K.K. & BLACKLEDGE, G.R.P. (0000). Intraperitoneal CA125: A potential tumour marker. *J. Tumor Marker Oncol.*, in press.

SCHILTHUIS, M.S., AALDERS, J.G., BOUMA, J. & 4 others (1987). Serum CA125 levels in epithelial ovarian cancer: Relation with findings at second-look operations and their role on the detection of tumour recurrence. *Br. J. Obstet. Gynaecol.*, **94**, 202.